# Food and nutrition of Gaur (*Bos gaurus* C.H. Smith, 1827) at the edge of Khao Yai National Park, Thailand

**DOI:** 10.1038/s41598-021-82858-1

**Published:** 2021-02-08

**Authors:** Rattanawat Chaiyarat, Suphat Prasopsin, Naris Bhumpakphan

**Affiliations:** 1 Wildlife and Plant Research Centre, Faculty of Environment and Resource Studies, Mahidol University, Nakhon Phathom, 73170 Nakhon Phathom Province Thailand; 2 Mahidol University, Kanchanaburi Campus, Sai Yok District, 71150 Kanchanaburi Province Thailand; 3 Department of Forest Biology, Faculty of Forestry, Kasetsart University, Chatuchak, Bangkok, 10900 Thailand

**Keywords:** Ecology, Zoology, Ecology, Environmental sciences

## Abstract

The presence of gaur (*Bos gaurus*) at the border of Khao Yai National Park (KYNP) in Thailand has resulted in a dramatic increase in the number of individuals’ crop feeding. This study examines the feeding adaptations of gaur at the edge of the protected area and assesses whether gaur response to increased nutrient availability in crop plants compared to natural forage. During the day, gaur mostly utilized forest areas in KYNP and entered the agricultural areas at night. Gaur ate 43 natural forage species. Natural forage species contain high levels of crude protein and lipid, but they are found in small quantities and scattered areas when compared to crop plants, especially *Zea mays* L., that are available in large quantity and are heavily foraged on by gaur. However, greater understanding of the electivity index and nutrition of forage species along the edge of the protected area can be used to reduce the gaur-human conflict by keeping gaur in KYNP. Reducing the large monoculture areas that is the food sources of gaur along the edge may reduce or prevent gaur leaving the park and can be applied to advance conservation actions.

## Introduction

Human-wildlife coexistence at the edge of protected areas can create problems that are referred to as human-wildlife conflicts^1^. In general, specialist species are more affected by habitat modification than are generalist species. Moreover, some species are able to change to forage on food species that are more readily available when their preferred forage species are scarce^2^, thereby using crops as an alternative food source. Some crops are attractive to wild animals and provide both energy and nutrition^3^. However, this subject is poorly studied, especially in the large bovids of tropical environments.

**Figure 1:**
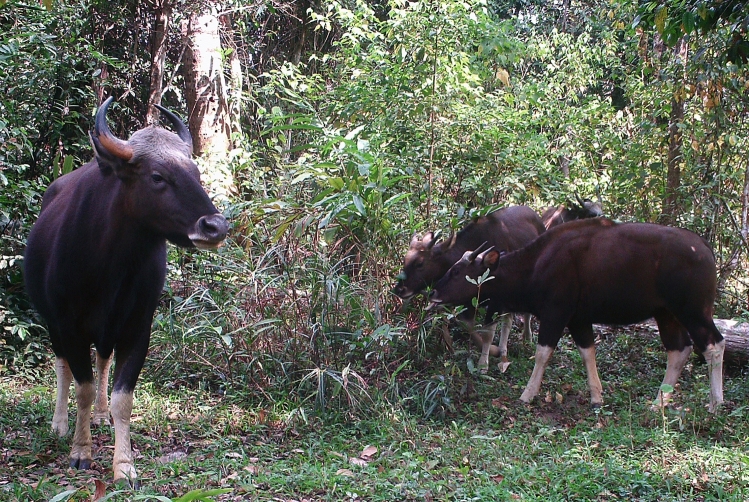
The gaur (*Bos gaurus*) are the largest extant bovids and feed at the edge of Khao Yai National Park, Thailand. Photographs from camera-trap by R. Chaiyarat.

Gaur (*Bos gaurus*), family Bovidae (Fig. 1), is globally vulnerable^4^, and protected under the Thai Reserved and Protected Animals Act, B.C.2562^5^. Gaur are distributed in scattered areas of Bhutan, Cambodia, China, India, Lao PDR, peninsular Malaysia, Myanmar, Nepal, Viet Nam and Thailand. The global population is estimated at 15,000–35,000 animals^4^. In Thailand, an estimated total of 920 Gaur remained in 1994 and none of them were found outside protected areas^6^, but a recent field survey found that the number of gaur are increasing^7^ and entering agricultural areas such as those near Khao Yai National Park (KYNP).

Gaur require a larger habitat area and higher food consumption than do smaller-bodied herbivores^8^. Based on the Jarman-Bell principle, the gaur’s large body mass allows it to subsist on lower quality forage than do smaller ruminants, but to meet their caloric demands, gaur require a larger quantity of it^9,10^. Gaur prefer grassland habitat and open areas of moist evergreen forest, dry evergreen forest, semi-evergreen forest, and mixed deciduous forest^11^. However, Steinmetz^12^ found that gaur are present in dry dipterocarp forest more often than in semi-evergreen forest. In the seasonal forest ecosystems of Thailand and India, gaur live in higher densities in mixed deciduous forests^13^ than in other available habitats^14^ due to the former having a richer array of available food types. This allows them to graze and browse at a single location^12^.

Gaur in Thailand are mainly restricted to protected areas because they are threatened by habitat loss and degradation, as documented in other areas^12,14–17^. When restricted to habitats in protected areas, the ground cover species used as forage, both monocots and dicots, are diminished in abundance. Then, gaur may move to disturbed and open areas along the border of protected areas^18,19^. Agricultural land that is accessible to gaur may be attractive because it meets their forage requirements. This may cause a positive association between gaur and human settlements. A number of studies found distance to protected edge as an important determinant in crop-raiding frequency by ungulates, suggesting that larger populations of wild ungulates lead to more agricultural damage near park edges^1^. The hypothesis that an increasing population of ungulates can cause considerable agricultural damage^20^ is widely believed, but it has never clearly been shown. Unfortunately, there are only a few studies on the food and nutrition of gaur living at the boundary between protected areas and agricultural areas. Crops, especially crop seeds, are high both in energy and palatablity but are low in protein^21^. In this study, we studied natural forage and crop species and nutrition to test the hypothesis that gaur were feeding more on highly nutritious crops grown outside the KYNP than on natural forage species. We investigate the feeding adaptations of gaur at the edge of a protected area and expect that gaur, *Bos gaurus,* caused crop damage in response to increased nutrient availability in crop plants compared to natural forage.

## Materials and methods

### Sample collection

All samples were taken from Khao Yai National Park with the permission from the Department of National Parks, Wildlife and Plant Conservation (DNP0907.1/11504). Research ethics, methods and experimental protocols were approved by the Mahidol University-Institute Animal Care and Use Committee (MU-IACUC 2017/022). Samples were collected and analyzed according to the guidelines of the ethics of wildlife research: A Nine R Theory^22^.

**Figure 2:**
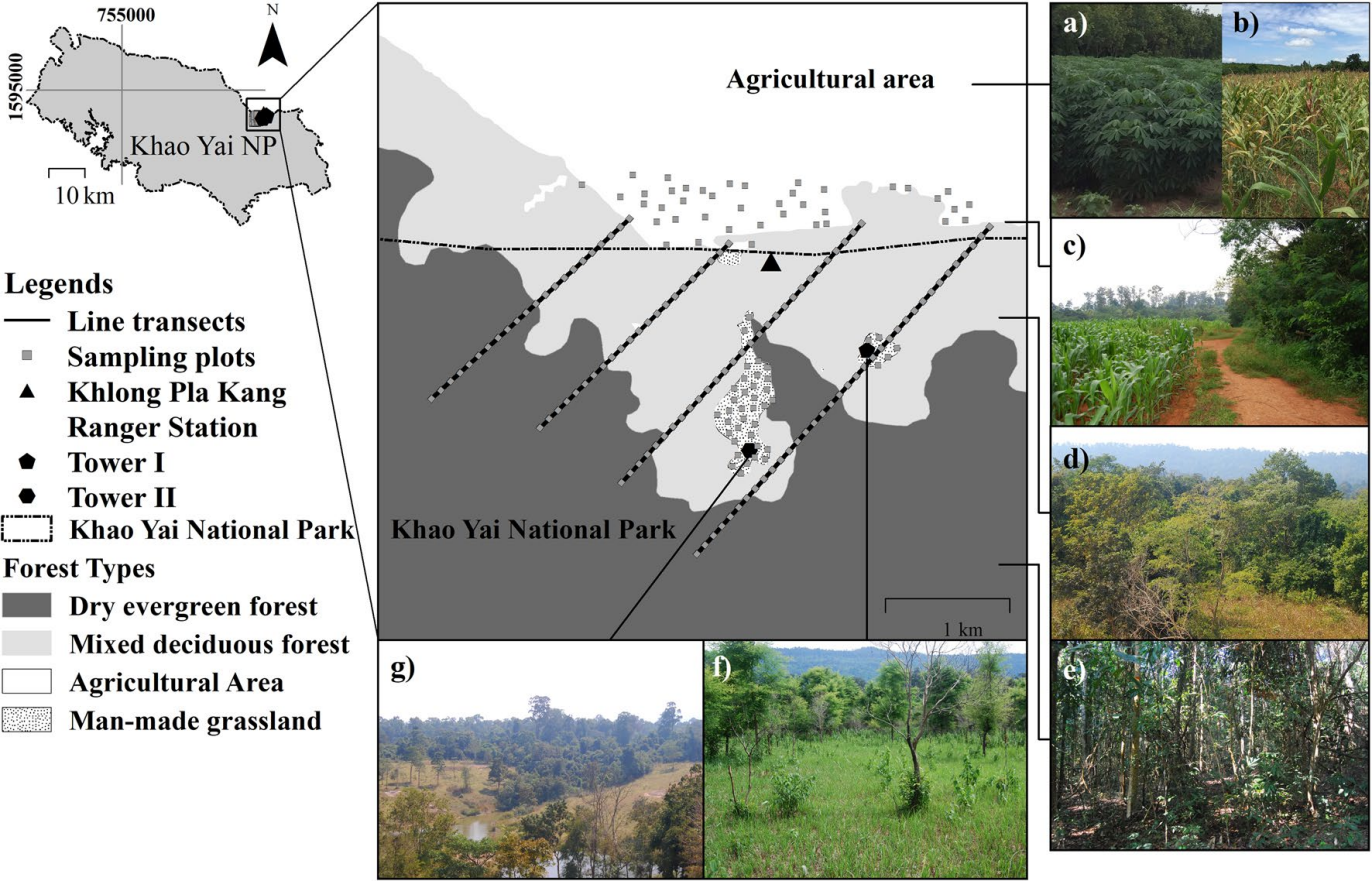
Study area and line transects of gaurs’ forage species and nutrition in the edge along Khlong Pla Kang National Park Guard Station, Khao Yai National Park. Maps created using ARC GIS 10.3, modified after Department of National Parks, Wildlife and Plant Conservation (DNP)^23^; (c and f) photographs by S. Prasopsin; all other photographs by R. Chaiyarat.

A study on the food and nutrition of gaur in KYNP was conducted in the disturbed edge between Khlong Pla Kang National Park Guard Station (KPK) and Khlong Pla Kang village (KPV), Nakhon Rachasima Province, Thailand between April 2009 and March 2010. Khao Yai National Park was the first national park designated in Thailand and has been listed as an ASEAN Heritage site in 2003 and a UNESCO World Heritage site in 2005. It is located at 14º05′–14°15′N and 101°05′–101°50′E with a total area of 2168 km^2^^23^. Khao Yai National Park is classified into seven forest types: hill evergreen forest (6.7%), moist evergreen forest (1.9%), mixed deciduous forest (24.2%), dry evergreen forest (62.1%), man-made grassland (1.4%), dry dipterocarp forest (0.5%) and secondary forest and other areas (3.1%)^24^. These areas are suitable for more than 71 mammal species such as dhole (*Cuon alpinus*), wild pig (*Sus scrofa*), sambar (*Rusa unicolor*), northern red muntjac (*Muntiacus vaginalis*) and gaur^25^, especially in the area around KPK. The forest types around KPK Station are mixed deciduous forest (48.4%), dry evergreen forest (49.6%), and man-made grassland (2%). Man-made grassland was part of the disturbance areas before KYNP was established and has been maintained as grass food sources for wildlife. The dominant species of trees are dipterocarpaceae. Furthermore, man-made grasslands are found in the abandoned agricultural areas, especially at the edges of the park which are frequently grazed by gaur (Fig. 2).

### Field surveys

Field surveys using binoculars (Nikon Aculon A211 8 × 42) were conducted to determine (a) the percentage of time gaur were observed in each type of forages at the following sites: (1) man-made grassland at wildlife observation tower number two, (2) man-made grassland at wildlife observation area number one, (3) the edge between KPK and the forest, and (4) an agricultural area outside the national park. These locations were chosen in order to compare activities across different habitat types. In each area, the percentage of gaur feeding time between 06:00 AM and 06:00 PM was recorded three days per month for 12 months. In the man-made grasslands, we used the wildlife observation towers, but in the forest areas, each location was selected based on natural signs indicating gaur activity, and we observed from blinds positioned to view the entire feeding area. The frequency of occurrence per area (%) was calculated from the proportion between the percentage of occurrence and percentage of the area. (b) the percentage of plant species on transect that had been grazed or browsed by gaur. Four transects with a total length of 21 km were located at the disturbed edge inside the protected area (natural forages) to study the gaurs’ food utilization and nutrition. A total of 210 plots (1 × 2 m^2^) were sampled along four transects at intervals of 100 m apart. Most of these sampling plots were located in dry evergreen forest and mixed deciduous forest. In the small paths of man-made grassland and agricultural areas, the areas were crossed with a few line plots. Then, 40 random plots were randomly located and measured in each area in both dry season (November to April) and wet season (May to October) since systematic transect plots at intervals of 100 m apart from each other were not possible (Fig. 2). All stems (i.e. availability) of each species and number of stems showing evidence of gaur herbivory (i.e. use) were counted and the measurements extrapolated to stems per hectare. All detections of a plant species across the plots were pooled as recommended by Lashley et al.^26^.

To avoid the mistake of misidentifying the foraging of gaur for that of other herbivores, the structure of the damage in remaining forage tissues and the foraging ecology of gaur and other wildlife to distinguish herbivory between gaur and other wildlife species as Lashley et al.^27^ recommended. This method was particularly useful in distinguishing between lagomorphs’ indirect bite and that of ungulates. However, distinguishing herbivory between wildlife species can be difficult and may result in sampling errors related to damage detectability that may change with leaf morphology of plant species. To limit false counts of gaur direct bites, only plants with signs of newly eaten vegetation, without necrotic black tissue surrounding the older bite^26,27^, after intensive consideration of the distinctive bite morphology along with new signs of gaur hoof prints in the area, without any other signs of other herbivore species, were recorded. At the disturbed edge area, only gaur, a small population of sambar deer (*Rusa unicolor*), and elephants (*Elephas maximus*) were directly observed. Other small nocturnal mammals (e.g. Northern Red Muntjac, *Muntiacus vaginalis*) or Greater Oriental Chevrotain (*Tragulus napu*)) were not found or had different forage species and bite behavior. These animals had no impact on the study.

### The electivity index of individual forage species consumed

At the end of each season, a twig-count method^28^ was used to measure the percentage of individual species forage biomass found in the areas. In each consumed species, the diameters of fifty fresh stems at the bite location were measured and averaged. Based on an average stem diameter, fifty fresh stems without bite signs beside the sample plots were clipped and weighed to the nearest 0.01 g. Samples of each plant species, both fresh and consumed, were separated, bagged in plastic and sent to Mahidol University Laboratory at Kanchanaburi Campus. At the laboratory, each sample was put in a small paper bag, and dried in an air-flow dryer at 50 °C^27^ until the weight was unchanged. The weight of each forage species was converted to the biomass for available and consumed in the areas.

The electivity index was calculated by dividing the ratio of use (i.e. stems browsed for bite count and the proportion of the diet for indirect count) and availability (i.e. total number of stems for bite count and available biomass for indirect count) for a given species by the sum of ratios for all species. The Ivlev electivity index^29^ of individual forage species consumed in the study plots was calculated as follows:

$$E_{i} = \left( {r_{i} {-}P_{i} } \right)/\left( {r_{i} + P_{i} } \right)$$

where *E*_*i*_ is the Ivlev electivity index, *r*_*i*_ is the relative removal in each forage species and *P*_*i*_ is the relative availability in each species in the ecosystem. *E*_*i*_ is scaled so that *E*_*i*_ = -1 corresponds to total avoidance of, *E*_*i*_ = 0 represents non-selective feeding on, and *E*_*i*_ = 1 shows exclusive feeding on a given forage species *i*.

### Food quality assessment

Ten samples of selected plants, including exclusive feeding and avoidance by gaur based on both high and low electivity indices, were collected for natural forage species (inside the protected area) and for agriculture crop plant species (agricultural area). Only new shoots consumed by gaur were selected as samples. All samples were selected in the wet season except for *Alstonia scholaris* (L.) R.Br. were selected in the dry season as the electivity index of this species was high in the dry season. Ten individual plants (100 g) per species were collected and sent to Mahidol University Laboratory at Kanchanaburi Campus for plant nutrient value analysis including the moisture content, ash, crude fiber, crude protein and lipid.

Moisture content was analyzed by oven drying at 105 °C for 16 hrs^30^. Ash was determined by ashing (Furance, model AWF12/42, Lenton, UK) at 600 °C for 2 hrs^31^. Fiber was analyzed by used fibertherm (model FT12, Gerhardt, Germany) measurement^32^. Crude protein was determined using Kjeldahl (model KB8, Gerhardt, Germany) nitrogen measurement^32^. Lipid was analyzed using Soxhlet (model S306AK, Gerhardt, Germany) procedures^33^.

### Statistical analysis

One-way ANOVA test was used to compare the number among forage species types (tree, shrub, herb, grass, climber, shrubby tree, exotic tree, scandent shrub, under shrub, exotic grass and woody climber) between wet (May to October) and dry (November to April) seasons at the edge using SPSS.

## Results

### Detect probabilities to compare observations across habitat types

The gaur grazed more in man-made grassland (43.3% of observations counted) than in other areas (Table 1). During day time, the relative occurrence per observation area was highest in man-made grassland (60.8% and 38.3%) and lowest in the forest between KPK and agricultural areas and where gaur entered agricultural areas at night.

**Table 1:** Numbers of gaur feeding at the edge along Khlong Pla Kang National Park Guard Station, Khao Yai National Park (n = 36 days).

Occurrence	Feeding site^a^			
	1	2	3	4
Number of occurrence (times)	16	52	35	17
Percentage of occurrence (%)	13.3	43.3	29.2	14.2
Observed area (km^2^)	0.06	0.31	18.5	7.7
Percentage of area (%)	0.2	1.2	69.6	29
Frequency of occurrence per area (%)	60.8	38.3	0.4	0.5

^a^(1) = Man-made grassland at wildlife observation tower number 2, (2) Man-made grassland at wildlife observation number 1, (3) Boundary between Khlong Pla Kang National Park Guard Station and agriculture area; this area also served as resting area before going to the agriculture area, and (4) agriculture area outside the national park.

### Forage species

A total of 43 natural forage species were recorded in the study area. As many as 41 of these species were consumed by gaur during the wet season, while 25 species were consumed in the dry season. Additionally, 23 species were consumed in both seasons (Tables 2 and 3). The electivity index of natural forage species showed that *Ageratum conyzoides* L. had the highest value in wet seasons and *Chromolaena odorata* (L.) R.M. King & H. Rob. had a high electivity index in both seasons. While crop plants such as *Manihot esculenta* Crantz had an exclusive electivity index in wet season, and *Zea mays* L. had an avoid electivity index in wet season, but it was highest in availability and consumption (Table 3). The gaur did not show a strong preference in forage species between dicots and monocots or between seasons (*F* = 0.976; *df* = 3, 18; *p* = 0.32), although dicots were more eaten by gaur in both wet (80.5%) and dry (84%) seasons (Table 4).

**Table 2:** Species list of gaurs’ forage species and their edible parts in the transition zone around Khlong Pla Kang National Park Guard Station, Khao Yai National Park.

Family	Scientific Name	Forage Species^a^	Eaten Part^b^	Habitat type^c^			
				DEF	FP	TGL	AA
**Natural forage species**							
Apocynaceae	*Alstonia scholaris* (L.) R.Br	T	L, S	N	P	P	A
Apocynaceae	*Wrightia arborea* (Dennst.) Mabb	T	L	P	P	A	A
Asteraceae	*Ageratum conyzoides* L	H	F, S	P	P	P	P
Asteraceae	*Chromolaena odorata* (L.) R.M. King & H. Rob	H	F, S	P	P	P	P
Bignoniaceae	*Markhamia stipulata* Seem	T	L	P	P	A	A
Combretaceae	*Combretum deciduum* Collet & Hemsl	S	L	P	P	A	A
Costaceae	*Costus speciosus* (Koen.) Sm	H	L	P	P	A	A
Cyperaceae	*Cyperus* sp.	Gs	L	A	A	P	A
Dilleniaceae	*Dillenia obovata* (Blume) Hoogland	T	L	A	P	A	A
Dioscoreaceae	*Dioscorea glabra* Roxb	C	L	A	P	P	A
Dipterocarpaceae	*Dipterocarpus turbinatus* C.F. Gaertn	T	L	P	A	A	A
Euphorbiaceae	*Croton persimilis* Mull. Arg	S	S	A	P	A	A
Fabaceae	*Acacia catechu* (L.f.) Willd	ExT	L	A	P	A	A
Fabaceae	*Acacia concinna* (willd.) DC	ScanS	L	A	P	P	A
Fabaceae	*Adenanthera pavonina* L	T	L	A	P	A	A
Fabaceae	*Pterocarpus macrocarpus* Kurz	T	L	P	P	A	A
Fabaeae	*Xylia xylocarpa* (Roxb.) Taub	T	L	P	P	A	A
Irvingiaceae	*Irvingia malayana* Oliv. Ex A.W.Benn	T	L	P	A	A	A
Lauraceae	*Cinnamomum iners* Reinw. Ex Blume	T	L	P	A	A	A
Lauraceae	*Litsea glutinosa* (Lour.) C.B.Rob	T	L	P	A	A	A
Malvaceae	*Helicteres lanata* (Teijsm. & Binn.) Kurz	S	L	P	P	P	A
Malvaceae	*Mallotus paniculatus* Mull. Arg	T	L, S	A	P	A	A
Malvaceae	*Mallotus philippensis* Mull. Arg	ST	L	A	P	P	A
Malvaceae	*Microcos paniculata* L	T	L	A	P	A	A
Malvaceae	*Urena lobata* L	US	L	A	P	P	P
Menispermaceae	*Tiliacora triandra* (Colebr.) Diels	H	L	P	P	A	A
Musaceae	*Musa cylindric* Colla	H	L, S	A	P	A	A
Oleaceae	*Jasminum anodontum* Gagnep	C	L	A	P	A	A
Oleaceae	*Jasminum simplicifolium* G. Forst	C	L	A	P	A	A
Phyllanthaeae	*Bischofia javanica* Blume	T	L	A	P	A	A
Phyllanthaceae	*Phyllanthus emblica* L	T	S	A	P	A	A
Phyllanthaceae	*Phyllanthus* sp.1	H	L	A	P	P	A
Phyllanthaceae	*Phyllanthus* sp.2	S	L, S	A	P	A	A
Poaceae	*Arundo donax* L	Gs	L	A	P	P	A
Poaceae	*Brachiaria mutica* (Forssk.) Stapf	Gs	L	A	A	P	A
Poaceae	*Imperata cylindrica* (L.) Raeusch	Gs	L	A	P	P	P
Poaceae	*Neyraudia reynaudiana* (Kunth) H.Keng ex Hitchc	Gs	L	A	P	P	P
Poaceae	*Pennisetum polystachyon* (L.) Schult	ExGs	L	A	P	P	P
Poaceae	*Sorghum halepense* (L.) Pers	Gs	L	A	P	P	P
Poaceae	*Sorghum propinquum* (Kunth) Hitchc	Gs	L	A	P	P	A
Rhamnaceae	*Ziziphus oenoplia* (L.) Mill	WC	L	A	P	P	A
Rubiaceae	*Ixora umbellate* Valeton ex koord	S	S	P	P	A	A
Simaroubaceae	*Harrisonia perforata* (Blanco) Merr	ScanS	L, S	A	P	P	A
**Agriculture crop plant**							
Annonaceae	*Annona squamosa* L. (Sugar apple)	ExST	L, S	A	A	A	P
Euphorbiaceae	*Hevea brasiliensis* Muell. Arg. (Pará rubber tree)	ExT	L, S	A	A	A	P
Euphorbiaceae	*Manihot esculenta* Crantz (Cassava)	ExS/ST	L, S	A	A	A	P
Myrtaceae	*Eucalyptus camaldulensis* Dehnh. (River red gum)	ExT	L, S	A	A	A	P
Poaceae	*Zea mays* L. (Corn)	ExGs	L, S	A	A	A	P
Solanaceae	*Capsicum annuum* L. (Capsicum)	ExS	L, S	A	A	A	P
Umbelliferae	*Coriandrum sativum* L. (Coriander)	ExH	L, S	A	A	A	P

^a^Forage species: C = Climber, ExGs = Exotic Grass, ExH = Exotic Herb, ExS = Exotic Shrub, ExS/ST = Exotic Shrub/Exotic Shrubby Tree, ExST = Exotic Shrubby Tree, ExT = Exotic Tree, Gs = Grass, H = Herb, S = Shrub, ScanS = Scandent Shrub, ST = Shrubby Tree, T = Tree, US = Under Shrub, WC = Woody Climber.

^b^Part of forage species: L = Leaf, F = Flower, S = Shoot.

**Table 3:** Biomass of available, removal and electivity index of gaurs’ forage species in transition zone around Khlong Pla Kang National Park Guard Station, Khao Yai National Park.

Family	Scientific name	Available (g ha^−1^)		Removal (g ha^−1^)		Electivity index	
		Dry	Wet	Dry	Wet	Dry	Wet
**Natural forage species**							
Apocynaceae	*Alstonia scholaris* (L.) R.Br	139.2	212	49.6	152.8	0.15	− 0.26
Apocynaceae	*Wrightia arborea* (Dennst.) Mabb	24.5	3.5	13.3	2.1	− 0.05	− 0.17
Asteraceae	*Ageratum conyzoides* L	N/A	28.8	N/A	0.2	N/A	0.97
Asteraceae	*Chromolaena odorata* (L.) R.M. King & H. Rob	170.6	454.8	4.5	11.1	0.9	0.89
Bignoniaceae	*Markhamia stipulata* Seem	N/A	14.5	N/A	3.5	N/A	0.28
Combretaceae	*Combretum deciduum* Collet & Hemsl	N/A	67.2	N/A	6.5	N/A	0.63
Costaceae	*Costus speciosus* (Koen.) Sm	N/A	1.4	N/A	0.4	N/A	0.2
Cyperaceae	*Cyperus* sp.	N/A	151.5	N/A	1.3	N/A	0.96
Dilleniaceae	*Dillenia obovata* (Blume) Hoogland	N/A	1.7	N/A	1.7	N/A	− 0.4
Dioscoreaceae	*Dioscorea glabra* Roxb	0.6	5.6	0.6	3.4	− 0.35	− 0.7
Dipterocarpaceae	*Dipterocarpus turbinatus* C.F. Gaertn	1.2	N/A	0.6	N/A	− 0.01	N/A
Euphorbiaceae	*Croton persimilis* Mull. Arg	N/A	1.3	N/A	0.5	N/A	0.05
Fabaceae	*Acacia catechu* (L.f.) Willd	N/A	209.1	N/A	108.8	N/A	− 0.1
Fabaceae	*Acacia concinna* (willd.) DC	73	125.8	40.5	88	− 0.07	− 0.24
Fabaceae	*Adenanthera pavonina* L	N/A	13.6	N/A	7.1	N/A	− 0.1
Fabaceae	*Pterocarpus macrocarpus* Kurz	4.6	21.5	0.6	16.8	0.58	− 0.29
Fabaeae	*Xylia xylocarpa* (Roxb.) Taub	N/A	9.3	N/A	2.3	N/A	0.27
Irvingiaceae	*Irvingia malayana* Oliv. Ex A.W.Benn	5.3	23.7	4.4	7.9	− 0.26	0.12
Lauraceae	*Cinnamomum iners* Reinw. Ex Blume	3.4	N/A	3.4	N/A	− 0.35	N/A
Lauraceae	*Litsea glutinosa* (Lour.) C.B.Rob	0.4	2.9	0.2	1.2	− 0.01	0.02
Malvaceae	*Helicteres lanata* (Teijsm. & Binn.) Kurz	N/A	0.7	N/A	0.7	N/A	− 0.4
Malvaceae	*Mallotus paniculatus* Mull. Arg	3	0.5	1.5	0.5	− 0.01	− 0.4
Malvaceae	*Mallotus philippensis* Mull. Arg	8.4	12.7	4.2	12.7	− 0.01	− 0.4
Malvaceae	*Microcos paniculata* L	N/A	100.4	N/A	56.4	N/A	− 0.14
Malvaceae	*Urena lobata* L	58	108.6	6.5	10.5	0.63	0.63
Menispermaceae	*Tiliacora triandra* (Colebr.) Diels	N/A	0.8	N/A	0.8	N/A	− 0.4
Musaceae	*Musa cylindric* Colla	3.4	2.3	2.4	0.5	− 0.18	0.33
Oleaceae	*Jasminum anodontum* Gagnep	11.4	52	6.8	47.1	− 0.1	− 0.36
Oleaceae	*Jasminum simplicifolium* G. Forst	25.2	26.5	14.6	15.6	− 0.09	− 0.16
Phyllanthaeae	*Bischofia javanica* Blume	N/A	8.4	N/A	6.4	N/A	− 0.28
Phyllanthaceae	*Phyllanthus emblica* L	N/A	38.4	N/A	25.6	N/A	− 0.22
Phyllanthaceae	*Phyllanthus* sp.1	4.3	12.5	1.7	4.3	0.1	0.11
Phyllanthaceae	*Phyllanthus* sp.2	2.4	10.8	2	9.6	− 0.26	− 0.35
Poaceae	*Arundo donax* L	56.4	50.9	3.4	26.5	0.78	− 0.1
Poaceae	*Brachiaria mutica* (Forssk.) Stapf	N/A	1.8	N/A	0.9	N/A	− 0.08
Poaceae	*Imperata cylindrica* (L.) Raeusch	N/A	3638.5	N/A	2099.1	N/A	− 0.15
Poaceae	*Neyraudia reynaudiana* (Kunth) H.Keng ex Hitchc	59.1	14.3	53.8	11.2	− 0.3	− 0.29
Poaceae	*Pennisetum polystachyon* (L.) Schult	N/A	38.7	N/A	20.3	N/A	0.1
Poaceae	*Sorghum halepense* (L.) Pers	16.8	26.9	4.9	11.8	0.25	− 0.01
Poaceae	*Sorghum propinquum* (Kunth) Hitchc	14.5	105.9	8.8	63.6	− 0.11	− 0.17
Rhamnaceae	*Ziziphus oenoplia* (L.) Mill	23	127.5	4.7	63.2	0.01	− 0.07
Rubiaceae	*Ixora umbellate* Valeton ex koord	5.5	21.3	2.4	10.3	0.05	− 0.06
Simaroubaceae	*Harrisonia perforata* (Blanco) Merr	229.8	499.6	162.2	385.7	− 0.18	− 0.29
**Agricultural crop plant**							
Annonaceae	*Annona squamosa* L. (Sugar apple)	N/A	65.2	N/A	16.6	N/A	0.25
Euphorbiaceae	*Hevea brasiliensis* Muell. Arg. (Pará rubber tree)	29.9	N/A	6.6	N/A	0.38	N/A
Euphorbiaceae	*Manihot esculenta* Crantz (Cassava)	264	264	198	105	− 0.21	0.04
Myrtaceae	*Eucalyptus camaldulensis* Dehnh. (River red gum)	N/A	240	N/A	60	N/A	0.26
Poaceae	*Zea mays* L. (Corn)	N/A	2084.8	N/A	912.1	N/A	− 0.01
Solanaceae	*Capsicum annuum* L. (Capsicum)	N/A	686	N/A	196	N/A	0.2
Umbelliferae	*Coriandrum sativum* L. (Coriander)	N/A	4075.5	N/A	1254	N/A	0.16

N/A, Not analyzed.

**Table 4:** The type of forage species in the wet season and dry season at the edge along Khlong Pla Kang National Park Guard Station, Khao Yai National Park.

Season	Forage species (species)											Percentage of selection (%)	
	T	S	H	G	C	St	Et	Ss	Us	Eg	Wc	Dicots	Monocots
Wet	13	5	6	7	3	1	3	2	1	1	1	80.5	17.4
Dry	8	2	3	4	3	1	0	2	1	0	1	84	14.8
F	0.976												
df	3, 18												
*p*-value	0.426												

T = Tree; S = Shrub; H = Herb; G = Grass; C = Climber; St = Shrubby tree; Et = Exotic tree; Ss = Scandent shrub; Us = Under shrub; Eg = Exotic grass; Wc = Woody climber.

The electivity index of crop damage showed that most crops were consumed in the wet season with a low electivity index. Only *Hevea brasiliensis* Muell. Arg. was consumed in wet season. *Manihot esculenta* Crantz was consumed in both seasons (Table 3).

### Food quality assessment

Thirteen species (11 wild species and two crop plants) that were noted during direct observation as forage species were sampled. Moisture content, ash, fiber, crude protein and lipid were different among the species (*p* < 0.05). The highest fiber containing plants were *Mallotus paniculatus* Mull. Arg. (0.789 ± 0.044 mg g^−1^), *Imperata cylindrica* (L.) Raeusch. (0.734 ± 0.054 mg g^−1^), *Manihot esculenta* Crantz (0.597 ± 0.139 mg g^−1^), and *Zea mays* L. (0.535 ± 0.007 mg g^−1^). The average fiber content of all plants tested was 0.464 ± 0.185 mg g^−1^. The plants containing the highest crude protein were *Wrightia arborea* (Dennst.) Mabb. (0.686 ± 0.009 mg g^−1^), *Jasminum anodontum* Gagnep. (0.392 ± 0.002 mg g^−1^), and *Ageratum conyzoides* L. (0.382 ± 0.266 mg g^−1^), while crop plants *Manihot esculenta* Crantz (0.11 ± 0.019 mg/g) and *Zea mays* L. (0.081 ± 0.002 mg g^−1^) contained lower crude protein than average (0.226 ± 0.214 mg g^−1^) (F = 11.842, df 12, 26, *p* < 0.001). Moreover, lipid content in *Alstonia scholaris* (L.) R.Br*.* (0.064 ± 0.006 mg g^−1^), *Wrightia arborea* (Dennst.) Mabb. (0.057 ± 0.002 mg g^−1^), *Mallotus paniculatus* Mull. Arg. (0.045 ± 0.004 mg g^−1^), *Chromolaena odorata* (L.) R.M. King & H. Rob. (0.035 ± 0003 mg g^−1^) and *Manihot esculenta* Crantz (0.039 ± 0.000 mg g^−1^) were higher than average (0.028 ± 0.019 mg g^−1^) (F = 111.28; df = 12, 26; *p* < 0.001) (Table 5).

**Table 5:** Food quality of gaur foods in the transition zone around Khlong Pla Kang National Park Guard Station, Khao Yai National Park (n = 3).

Scientific name	Moisture content (%)^a^	Food quality (mg g^−1^)				Electivity index	
		Ash	Fiber	Crude protein	Lipid	Dry	Wet
**Natural forage species**							
*Chromolaena odorata* (L.) R.M. King & H. Rob	54.17^b^	0.097 ± 0.001^d^	0.277 ± 0.01^ab^	0.101 ± 0.001^ab^	0.035 ± 0.003^d^	0.9	0.89
*Ageratum conyzoides* L	44.54^b^	0.096 ± 0.001^d^	0.359 ± 0.009^b^	0.382 ± 0.266^d^	0.013 ± 0.005^abc^	N/A	0.97
*Alstonia scholaris* (L.) R.Br	68^c^	0.154 ± 0.001f.	0.466 ± 0.091^bc^	0.06 ± 0.001^a^	0.064 ± 0.006f.	0.15	− 0.26
*Croton persimilis* Mull. Arg	46.43^b^	0.106 ± 0.001^e^	0.388 ± 0.005^b^	0.083 ± 0.001^a^	0.02 ± 0.001^c^	N/A	0.05
*Mallotus paniculatus* Mull. Arg	63.64^c^	0.08 ± 0.001^c^	0.789 ± 0.044^d^	0.229 ± 0.22^bc^	0.045 ± 0.004^e^	− 0.01	− 0.4
*Wrightia arborea* (Dennst.) Mabb	36.36^b^	0.092 ± 0.000^d^	0.349 ± 0.001^b^	0.686 ± 0.009^e^	0.057 ± 0.002f.	− 0.05	− 0.17
*Jasminum anodontum* Gagnep	40.41^b^	0.057 ± 0.001^b^	0.339 ± 0.007^b^	0.392 ± 0.002^d^	0.014 ± 0.003^abc^	− 0.1	− 0.36
*Microcos paniculata* L	34.02^b^	0.058 ± 0.004^b^	0.071 ± 0.089^a^	0.119 ± 0.001^ab^	0.008 ± 0.001^ab^	N/A	− 0.14
*Imperata cylindrica* (L.) Raeusch	14.71^a^	0.059 ± 0.001^b^	0.734 ± 0.054^d^	0.036 ± 0.007^a^	0.018 ± 0.001^bc^	N/A	− 0.15
*Harrisonia perforata* (Blanco) Merr	47.22^b^	0.057 ± 0.001^b^	0.364 ± 0.07^b^	0.075 ± 0.001^ab^	0.006 ± 0.003^a^	− 0.18	− 0.29
*Phyllanthus emblica* L	19.8^a^	0.027 ± 0.004^a^	0.332 ± 0.003^b^	0.328 ± 0.006^d^	0.023 ± 0.004^c^	N/A	− 0.22
Mean ± s.d	42.66 ± 15.84	0.08 ± 0.033	0.464 ± 0.185	0.226 ± 0.214	0.028 ± 0.019		
**Agriculture crop plant**							
*Manihot esculenta* Crantz	68.48 ± 6.48^c^	0.123 ± 0.002f.	0.597 ± 0.139^c^	0.11 ± 0.019^ab^	0.039 ± 0.000^de^	− 0.21	0.04
*Zea mays* L	73.67 ± 3.4^c^	0.119 ± 0.002f.	0.535 ± 0.007^c^	0.081 ± 0.002^ab^	0.01 ± 0.004^ab^	N/A	− 0.01
F	246.331	1094.632	2.404	11.842	101.016		
df	12, 26	12, 26	12, 26	12, 26	12, 26		
*p*-value	< 0.001	< 0.001	0.025	< 0.001	< 0.001		

(mean + sd), a = sd < 0.001, N/A = not analyzed due to not found consumed.

## Discussion

Feeding ecology is an important aspect of understanding the relationships between consumers and their environments^34^ in order to explain the increase in crop raids at the edge of protected areas. Using indirect bite count surveys to calculate the preferences for individual plant species has flaws. We suggest that in diet selection studies, it is preferable to use microhistological surveys together with indirect bite counts to assess the utility of the latter in gaur diet selection (see Holechek et al.^35^ and Lashley et al.^26^ for comparative approaches).

In KPK, gaur sightings occurred mostly in man-made grassland during the day time (06:00 AM and 06.00 PM). Gaur have previously been reported in Northern Kerala, India to enter grassland areas during the night time (06:00 PM–06:00 AM) (Jayson, 2016)^36^. The forage species selected by gaur did not different between dicots and monocots in both dry and wet seasons. These results differed from those reported by Bidayabha^18^ on the gaur population in the Khao Phaeng-Ma Non-Hunting Area (KPM) adjacent to KYNP. The KPK is mainly covered by evergreen and deciduous forests, but the KPM Area was restored mainly from man-made grassland and in 2017 Prayong and Srikosamatara^37^ found forage grass species in KPM were covered by pioneer tree species. There can be large changes in wildlife intake on grassland over different periods^38,39^. In this way, wildlife can show an opportunistic behavior in relation to forage availability^40^. The differences between browsers and grazers extend beyond diet selection; they include specialization within the digestive tract that may allow grazing and browsing herbivores to better extract nutrients from their preferred forage class (grass or browse)^41,42^. Ungulate species have been found to have different food and feeding habits^43^. Gaur, have been described as grazers^42,44,45^, browsers^46^ and generalists^47^ depending on habitat types.

Additionally, the results of our study show that 43 species of plants are consumed by gaur in KPK. Eighteen forage species were absent in the dry season. A change of food preference by animals during the vegetative growth season was clearly pointed out by Fresehi et al.^48^, and this could be due to modification in palatability of forage species according to their different stage of development^39^. It could also be influenced by forage biomass or plants’ reaction to utilization, and that reaction can change during growing seasons^49^. In the natural habitat, this process is important in ecology^50^ and should be accounted for in future studies. This evolution cannot be negative because the utilization of native species by wildlife can occur to a remarkable degree, and species can adapt, in particular situations, to browse on species of reduced forage quality^38^.

The variation in preference rating could be influenced by difference in the plant species and mode of presentation^51^.

For example, the electivity index of natural forage species showed that *Ageratum conyzoides* L. had the highest value in wet seasons, *Chromolaena odorata* (L.) R.M. King & H. Rob. had high electivity index in both seasons. These two species are high in crude protein that induce gaur to select them with a high electivity index, even though *Ageratum conyzoides* L. was scare and low in biomass per area when compared to other species. *Chromolaena odorata* (L.) R.M. King & H. Rob. is available or present in all habitat types, but it emits strong odors and may not be favorable or relished if gaur have other forage choices as explained by Kaitho et al.^52^. In comparison, *Wrightia arborea* (Dennst.) Mabb. contains the highest crude protein, but it produces a white latex that may not be favorable to gaur. Furthermore, the texture and chemical constituents of the leaves^51^, secondary compounds, macronutrient concentrations, flavors and odors^53,54^ also have been found to be important to preference rating of animals. These factors are not taken into account in this study and are recommended for future study. It should be noted that crop plants such as *Manihot esculenta* Crantz and *Zea mays* L. are higher in moisture content and are grown in larger areas as a monoculture as opposed to natural forage that is usually scattered in small areas. Additionally, crude protein in the *Manihot esculenta* Crantz and *Zea mays* L. were lower than the average of wild forage species, while lipids were higher than the average of natural forage species. These lipids can give gaur more energy to support their activities in shorter feedings within a large area of crop plants. This finding can support the theory of crop feeding as an optimizing strategy in which gaur choose behavioral strategies that are most likely to give them maximum benefit in comparison with the cost incurred^55^. Gaur may encounter the dangers from agricultural owners when feeding their crops.

Our results at the edge of KPK indicated that gaur were entering the agriculture areas at higher rates in the wet season (May to October). Because most crop plants are dependent on rainfall^36,50^, they are grown in the wet season and harvested in the dry season. The mineral content in consumed species (> 80% dicots) was higher in KYNP than in gaur dietary items in Bhagvan Mahaveer Wildlife Sanctuary and Mollem National Park, India (> 60% monocots)^16^ presumably due to different geographical and variation in the plant community (more open area than KPK). In spite of the forage quality of crop plants such as *Manihot esculent*a Crantz and *Zea mays* L. In early the wet season, the quality of crop plants are nearly equal when compared to natural species, but late in the wet season, the quality of natural species were dropped off much faster than crop plants. During this period, the availability of crop plants at the edge of KPK may have contributed to the crop raiding and gaur-human conflict in the area, since the forage availability in KYNP has sparse amounts of low herbaceous ground cover and grass that are the main forage species of gaur^56^. Retamosa et al.^57^ suggested that reliance on crops will be increased when crop plants such as *Zea mays* L. are located close to the woodland as found at the edge of KPK.

The primary conservation technique used at the edge was managing grasslands to improve the quantity and quality of grasses and other food plants for successful and sustainable conservation of gaur. The critical problem is to have cooperation of local residents to mitigate conflicts of interest^58^ between conservation and economics. Improving public awareness by conducting outreach programs and strict law enforcement by patrolling combined with habitat management along the habitat site might reduce human and gaur conflict in the area.

This study suggests that gaur living in edge areas are generalists and consume forage species as opportunists. Gaur entered the agricultural areas at the edge of the protected area, even though the food quality of crop species was lower than the average of natural forage species. *Manihot esculent*a Crantz is the main crop damaged in the area as they are high in crude protein and lipid. Growing crops in the large areas beside the protected area in the wet season will induce gaur to move to these areas and can increase gaur-human conflict in the future. Finally, this research may be used to improve knowledge on gaur feeding behavior and food quality. Which have relevance for future-planned management and conservation to improve the habitat quality of the gaur population in the areas and reduce the large areas of monocrop around the edge of the protected areas to improve farmer vigilance and increase benefits for farmers who have to live next to this protected area.
